# Relationship between children with neurodevelopmental disorders and their caregivers and friends during early phase of COVID-19 school closure in Japan: Association with difficulty in implementing infection prevention measures

**DOI:** 10.1186/s13034-022-00513-7

**Published:** 2022-10-07

**Authors:** Tomoka Yamamoto, Sanae Tanaka, Arika Yoshizaki, Yuko Yoshimura, Aishah Ahmad Fauzi, Aida Syarinaz, Ahmad Adlan, Subhashini Jayanath, Norhamizan Hamzah, Haruo Fujino, Masaya Tachibana

**Affiliations:** 1 Molecular research center for child development, United Graduate School of Child Development, Osaka University, 2-2 Yamadaoka, 565-0871 Suita, Osaka Japan; 2 Research Center for Child Mental Development, Kanazawa University, 13-1 Takaramachi, 920-8640 Kanazawa, Ishikawa Japan; 3 Department of Rehabilitation Medicine, Faculty of Medicine, University of Malaya, 50603 Kuala Lumpur, Malaysia; 4 Department of Psychological Medicine, Faculty of Medicine, University of Malaya, 50603 Kuala Lumpur, Malaysia; 5 Department of Pediatrics, Faculty of Medicine, University of Malaya, 50603 Kuala Lumpur, Malaysia; 6 United Graduate School of Child Development, Osaka University , 2-2 Yamadaoka, 565-0871 Suita, Osaka, Japan

**Keywords:** Infection prevention measures, Children with neurodevelopmental disorders, Relationship deterioration, School reopening

## Abstract

**Background:**

Due to the COVID-19 pandemic people had to implement various infection prevention measures. Researchers have reported the difficulties experienced by children with neurodevelopmental disorders in implementing these measures and their caregivers’ resultant anxiety and stress. This study examined the relationship between these difficulties and the deterioration of the children’s relationships with their caregivers and friends during school closure and after school reopened.

**Methods:**

A total of 150 caregivers of children with neurodevelopmental disorders answered a questionnaire asking about parent‒child relationships, their child’s friendships, and the presence or absence of difficulty in implementing infection prevention measures at three time points: before the pandemic, while schools were closed, and after school reopened. The frequency and percentages of the child’s behavioral problems, deterioration in their relationships, and difficulty implementing infection control measures were calculated. Using the relationship deterioration scores, independent and multiple regression analyses were performed for the presence or absence of difficulty implementing infection control measures, presence or absence of caregivers’ mental health concerns, and the presence or absence of deterioration of one or more problematic behaviors.

**Results:**

Overall, 84.1% of the children displayed difficulties implementing infection prevention measures. No relationship was observed between difficulty with infection prevention measures and deterioration in their relationships with parents and friends when schools were closed. After school reopened, however, deterioration in parent‒child relationships correlated positively with difficulty in hand-washing, and deterioration of friendships correlated positively with the maintenance of social distancing and difficulty in hand-washing. Deterioration of friendships correlated negatively with difficulty in voluntarily complying with stay-at-home requests.

**Conclusion:**

Difficulty in implementing infection prevention measures was related to deterioration in social relationships with parents and friends of children with neurodevelopmental disorders during the school reopening period, following COVID-19 school closure in Japan. Under a condition requiring heightened infection control, close monitoring may be necessary for the social relationships in children with neurodevelopmental disorders.

**Supplementary information:**

The online version contains supplementary material available at 10.1186/s13034-022-00513-7.

## Background

With the spread of COVID-19, many countries imposed nationwide lockdowns, restricting social activities. Although no official lockdowns were imposed in Japan, schools closed on February 27, 2020. After a state of emergency was declared on April 17, people were requested to stay at home voluntarily, and large-scale retail outlets were mandated to shut down. Over 25% of people experienced working from home [1], causing a dramatic change in the living environment of children and their families. Most schools reopened on May 25, 2020, and children began attending school again. Since then, while infection control measures such as frequent washing of hands, airing out living spaces, avoiding close proximity, and wearing masks when in close proximity have been recommended, no further nationwide school closure measures have been taken.

Neurodevelopmental disorders are brain dysfunctions observed since childhood, and include autism spectrum disorder (ASD), attention deficit hyperactivity disorder (ADHD), and intellectual disability (ID). In children, an association has been found between neurodevelopmental disorders and the child’s relationships with people. For example, they face the risk of deteriorated parent‒child relationships [2, 3] and difficulties in their relationships with friends [4].

These problems worsened during the COVID-19 pandemic. Caregivers of children with ASD reportedly faced increased stress during lockdowns and their relationships with the children in their care deteriorated [5]. Moreover, children with ASD and ADHD faced greater difficulties in their peer relationships during the initial phase of the COVID-19 spread [6]. In Japan, where stringent regulations were not imposed, caregivers of children with neurodevelopmental disorders experienced deteriorating mental health [7], and changes were observed in the relationships between children with neurotypical development and their friends [8]. However, the impact of environmental changes caused by the COVID-19 crisis on the deterioration of relationships with others has not been fully investigated, especially among neurodivergent people.

Studies conducted before the COVID-19 pandemic have indicated that various factors influence the deterioration of relationships with parents and friends among children with neurodevelopmental disorders. Regarding parent‒child relationships, researchers have observed associations between externalizing behavior problems [9] and the risk of abuse due to the presence or absence of ASD, ADHD, and ID [2, 10]. During the COVID-19 pandemic, in addition to lack of social support [11], caregivers’ nursing care burden [12], and parents’ increased stress were reported to be linked to the deterioration of parent‒child relationships [5]. Relationships with friends have been reported to worsen if a child has neurodevelopmental disorders, such as a diagnosis of ASD or ADHD [13], and in the presence of a child’s externalizing behavior problems [14]. Moreover, conduct problems have been associated with reduced online involvement with friends [15], special educational needs, the mother’s physical and mental ailments, and socioeconomic status [16], which are related to deteriorating relationships with friends during the COVID-19 pandemic.

During the pandemic, infection control measures must be practiced in daily life. In Japan, to prevent the spread of infection, the government promoted various control measures, such as staying at home, wearing masks when indoors or speaking to others, maintaining a two-meter distance from other people (hereafter, “social distancing”), washing hands frequently, and airing out rooms often [17]. However, children with neurodevelopmental disorders may find implementing infection control measures challenging because of their unwillingness to accept changes in routine and sensory characteristics. This not only causes stress in the children [18], but is reported to be associated with caregivers’ anxiety about becoming infected, causing stress to parents [19]. Difficulty implementing infection control measures was a factor influencing the high scores for depression, anxiety, and stress in caregivers of children with ASD [20], affecting interpersonal relationships.

This study evaluated changes in the behaviors and social relationships of children with neurodevelopmental disorders during the school closures following school closure due to the COVID-19 outbreak. It describes their difficulty implementing infection control measures, behavioral problems, and changes in their relationships with parents and friends, and investigates the factors inducing changes in such relationships.

## Methods

This study aimed to examine the relationship between the difficulties experienced by children with neurodevelopmental disorders in implementing infection prevention measures and the deterioration of their relationships with their caregivers and friends.

### Participants and survey period

Study participants were recruited at Osaka University Hospital’s Center for Developmental Medicine and Child Psychiatry (Pediatric Development Outpatient Clinic) and the Kanazawa University Research Center for Child Mental Development. We recruited 150 caregivers of children with neurodevelopmental disorders attending elementary or middle school as of October 2020 (aged 6–15 years). The survey period was October–December 2020. The study was approved by the Osaka University Hospital’s international review board, and written consent was obtained from the parents.

### Measurements

With the University of Malaya’s cooperation, we created an original questionnaire focusing on the participants’ concerns and their families’ needs during the COVID-19 pandemic [21] [see Additional file 1]. In addition to sociodemographic data (including age, sex, diagnosis, school, and family members) and the circumstances of the child and family when schools were closed (parents’ work status, limitations imposed during the state of emergency declaration, presence or absence of infections, presence or absence of the child’s involvement with their friends, presence or absence of caregivers’ mental health concerns, and presence or absence of social support offered), the child’s relationships, behavioral problems, and difficulties implementing infection control measures were also evaluated.

#### The child’s relationships

The participants were asked about their child’s relationship with others at three time points: before the spread of infection (January 2020), when schools were closed (April 17‒May 7, 2020), and after schools reopened (June 2020).

The following questions were asked about their child’s relationships with friends, parents, and siblings: “How was your child’s relationship with parents and friends before the pandemic, during the state of emergency declaration, and after schools reopened?” The answers were rated on a five-point scale: “Good,” “Fairly good,” “Neither good nor poor,” “Fairly poor,” or “Poor.”

#### The child’s behavioral problems

Questions about the child’s behavioral problems at three time points were evaluated to reveal the symptoms of neurodevelopmental disorders and the deterioration of secondary disorders. Fourteen problem-related items were established: hyperactivity, carelessness, social withdrawal, stereotyped speaking patterns, obsession, repetitive behaviors, sleep problems, rebellious behavior, food refusal, irritability, aggression, tantrums, self-injury, and school non-attendance. For each item, they were asked whether the behavior occurred before the pandemic and whether there was any worsening or new onset of these behavioral problems during the school closure period or after schools reopened. The participants were asked to choose items applicable to their children.

#### Difficulty implementing infection control measures

Questions about infection control were measured on the basis of infection countermeasures proposed in Japan as the “new normal.” These comprised six items: “Social distancing,” “Washing hands thoroughly after arriving home,” “Wearing a mask when going out,” “Taking one’s temperature and checking one’s health status every morning,” “Frequently airing out living spaces,” and “Voluntarily complying with stay-at-home requests.” The participants were asked to mention infection control measures that the child found challenging to implement since the spread of the infection. Multiple answers were accepted.

### Statistical analysis

Statistical analysis was conducted using JMP Pro 15.2.0. We calculated the frequency and percentages of the child’s behavioral problems, deterioration in their relationships, and difficulty implementing infection control measures. The Wilcoxon signed-rank test was used to analyze changes in relationships with others from before the pandemic to during school closure, and from school closure to after school reopening.

To investigate the factors related to deterioration in the child’s relationship with parents or friends, the objective variable of changes in scores in the deterioration of relationships with parents or friends was used. The scores for relationships with parents or friends before the pandemic were considered as the baseline, from which the difference in the scores when schools were closed was calculated to determine “scores for deterioration in relationships with parents or friends while schools were closed.” Thereafter, the scores for relationship with parents or friends while schools were closed were considered the baseline, and the difference in these scores after schools had reopened was calculated to determine the deterioration in relationships after schools reopened. The more the relationship deteriorated, the higher the deterioration scores. The deterioration scores of relationships with others were considered as the objective variable, and for each of the six types, independent and multiple regression analyses were performed for the presence or absence of difficulty implementing infection control measures, presence or absence of caregivers’ mental health concerns, and the presence or absence of deterioration of one or more problematic behaviors. In the multiple regression analysis, adjustments were made for age and sex. All continuous variables used for analysis were centralized and analyzed.

We also analyzed using a sample without children who do not attend school to examine whether school non-attendance may affect the results of the relationships, because the amount of time spent with parents or friends would be affected by school non-attendance.

## Results

### Sociodemographic and clinical characteristics

**Fig. 1:**
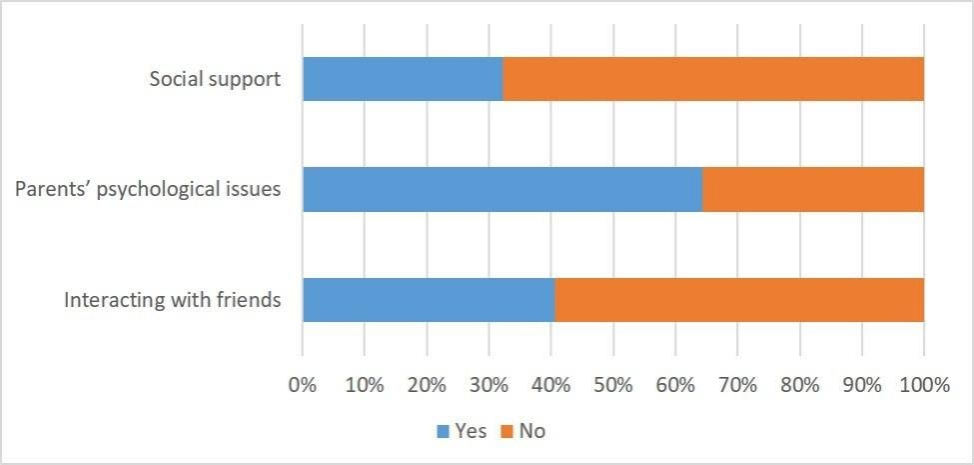
The situation during the spread of COVID-19

Of the 150 participants, 133 provided answers. Table 1 shows the children’s demographic data. The majority of children were diagnosed solely with ASD (50.0%), followed by those diagnosed with ADHD concomitantly with ASD (16.1%). Twelve children (9.1%) were not attending school before the infection spread. While the state of emergency was in force, 59.4% of the children did not interact in any way with their friends, 64.4% of the caregivers experienced some psychological problems, and only 32.3% of the caregivers received social support (Fig. 1; Table 2).

**Table 1:** Children’s demographic data

	Total number of responses	N	%
Child: Sex	133		
Male		104	78.2
Female		29	21.8
Child’s age, Mean (SD)	133	9.92	2.3 (SD)
Number of caregivers	132		
Single caregiver		25	18.9
Two caregivers		107	81.1
Number of sibling(s)	133		
0		36	27.1
1		68	51.1
2		26	19.6
3		3	2.3
Sibling(s) with disabilities		37	27.8
Fathers’ work status	125		
Employed		123	98.4
Changes in work patterns		47	37.6
Mothers’ work status	131		
Employed		79	60.3
Changes in work patterns		32	24.4
Diagnosis of neurodevelopmental disorders	128		
ASD^*^		64	50.0
ASD and ADHD		21	16.4
ASD and other neurodevelopmental disorders		14	10.9
ADHD		10	7.8
ASD and ADHD and other neurodevelopmental disorders		7	5.5
ADHD and other neurodevelopmental disorders		3	2.3
^a^Other neurodevelopmental disorders		3	2.3
School non-attendance before the spread of infection	132	12	9.1

^a^Including intellectual disability, learning disability, and other neurological diseases

^*^ASD: autism spectrum disorder, ADHD: attention deficit hyperactivity disorder

**Table 2:** The situation during the spread of COVID-19

	Total number of responses	N	%
Restrictions or voluntary stay-at-home during the state of emergency	132		
Going to school		124	93.9
Going shopping		105	79.6
Playing or exercising outside		91	68.9
Non-urgent medical care		72	54.6
Outdoor recreational activities		62	47.0
Going to an intervention center		40	30.3
Regular routine		36	27.3
Emergency medical care travel		6	4.6
Interacting with friends	133		
Yes		54	40.6
Parents’ psychological concerns	132		
Yes		85	64.4
Social support	130		
Yes		42	32.3

### Descriptive statistics of behavioral problems, relationships, and difficulty with infection control measures

#### Children’s behavioral problems

Of the 14 behavior problems items, one or more were observed in 60 children (45.8%) while schools were closed, and the average was 1.18 problems. Irritability was the most common behavioral problem observed in 37 children (28.0%). This was followed by sleep issues (18.9%) and tantrums (18.9%).

Meanwhile, 52 children (39.4%) showed one or more behavioral concerns after schools reopened, and the average was 1.18 problems. Irritability was the most common behavioral problem, observed in 27 children (20.5%). This was followed by rebellious behavior in 24 children (18.2%) and tantrums in 19 children (14.4%) (Table 3).

**Table 3:** Changes in children’s behavior problems

	N = 132		N = 132	
	Yes (n)	%	Yes (n)	%
	When schools were closed		After school reopened	
Irritability	37	28.03	27	20.45
Sleep problem	25	18.94	16	12.12
Tantrums	25	18.94	19	14.39
Rebellious behavior	18	13.64	24	18.18
Aggression	14	10.61	11	8.33
Obsession	12	10.61	13	9.85
Carelessness	6	4.55	11	8.33
Hyperactivity	5	3.79	6	4.55
Self-injury	4	3.03	2	1.52
Repetitive behavior	4	3.03	4	3.03
School non-attendance	4*	3.03	9	6.82
Stereotyped speaking pattern	3	2.27	3	2.27
Social withdrawing	1	0.76	2	1.52
Food refusal	0	0	1	0.76

* In Japan, some classrooms were open during the school closure because some of the children had no one to take care of them when their parents were out at work

#### Changes in relationships with others

**Fig. 2:**
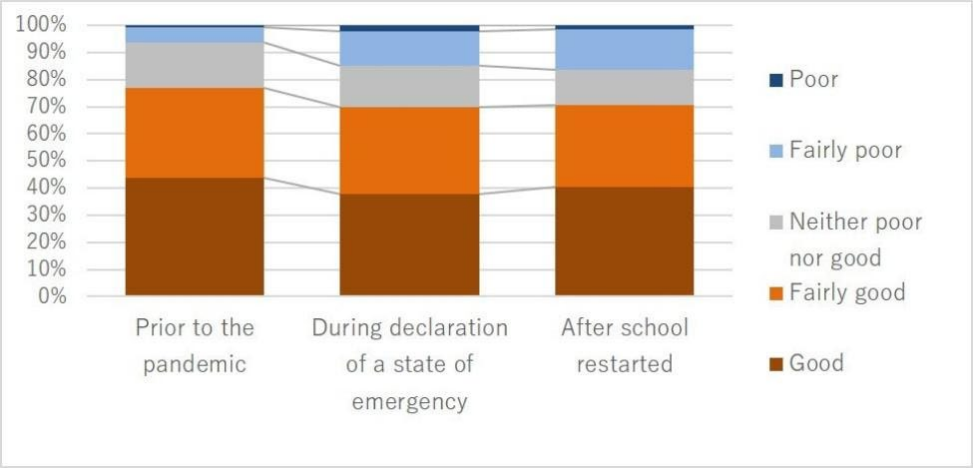
Changes in parent‒child relationships

The participants were asked to answer questions about their child’s relationships with friends and parents on a five-point scale at three time points, namely, before the pandemic, while schools were closed, and after schools reopened. Parent–child relationship scores were significantly worse when schools were closed than before the pandemic (S = 958.50, p = 0.004). However, there were no significant difference in scores from school closures to after school reopening (S = 60.50, p = 0.865). Deterioration scores of the parent‒child relationship while schools were closed showed an average increase of 0.22 points (SD = 0.90). Parent‒child relationships improved from before the pandemic for ten participants (7.8%), but deteriorated for 27 (20.9%). The deterioration scores of parent‒child relationships decreased by 0.02 points (SD = 0.85) after schools reopened compared with when schools were closed. Parent‒child relationships improved for 20 participants (15.5%) after schools reopened compared with when schools were closed, but deteriorated for 22 (17.1%) (Fig. 2).

**Fig. 3:**
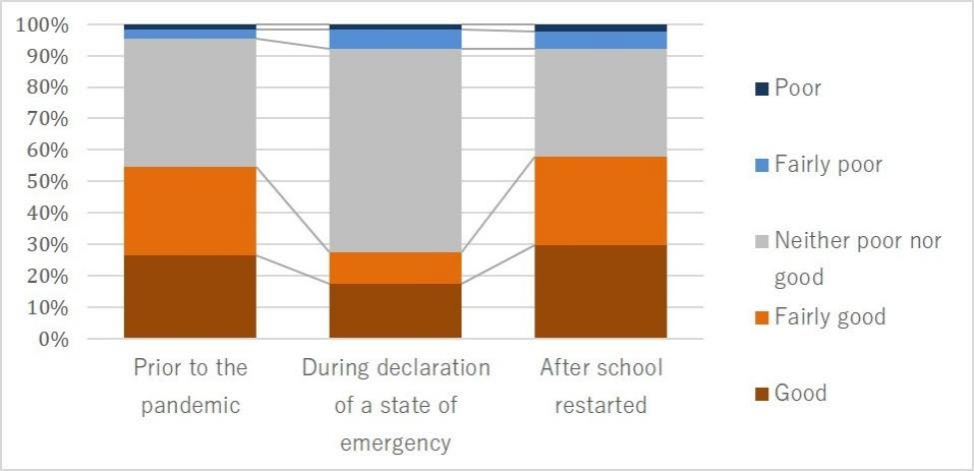
Changes in relationship with friends

The scores for children’s relationships with friends were significantly worse during school closure than before the pandemic (S = 2052.00, p < 0.001), while significant improvement was observed after school reopening (S=-1896.50, p < 0.001). The scores for deterioration of relationships with friends when schools were closed increased by an average of 0.40 points (SD = 0.75). Relationships with friends improved compared with before the pandemic for four participants (3.2%), but deteriorated for 44 (34.7%). Scores for deterioration of relationships with friends after schools reopened decreased by an average of 0.43 points (SD = 0.87) compared with when schools were closed. Relationships with friends improved for 45 participants (35.4%), but deteriorated for eight (6.3%) (Fig. 3).

The same changes in parent–child and friendship relationships were observed when children who did not attend school before the spread of infection were excluded.

#### Difficulty with infection control measures

A total of 111 caregivers (84.1%) experienced concerns regarding their children’s infection control measures. Social distancing was the most common infection control measure that was difficult to implement according to 54 participants (40.9%), followed by washing hands according to 38 (28.8%) and wearing a mask according to 35 (26.5%) (Table 4).

**Table 4:** Infection control measures found difficult by children

	N = 132	
	Yes (n)	%
Any of the following observed	111	84.09
Social distancing	54	40.91
Washing hands thoroughly when arriving home (Washing hands)	38	28.79
Wearing a mask indoors, outdoors, or when having a conversation (Mask)	35	26.52
Taking temperature and checking health status every morning (Health check)	31	23.48
Frequently airing out living spaces (Airing out)	23	17.42
Voluntarily complying with stay-at-home requests	22	16.67
Others	9	6.82

### Relationship between deterioration of relations with others and difficulty implementing infection control measures

Multiple regression analyses employing the forced entry method were performed using the following four deterioration scores as objective variables: parent–child relationships while schools were closed, parent‒child relationships after schools reopened, the child’s relationships with friends while schools were closed, and the child’s relationships with friends after schools had reopened. Age, sex, presence or absence of caregivers’ mental health problems, presence or absence of problematic behaviors, and presence or absence of difficulty implementing the six infection control measures were considered the explanatory variables. The results are shown in Tables 5 and 6. We examined the normality of the residuals in the quantile-quantile (QQ) plot but saw no significant deviations. All variance inflation factors (VIFs) were below ten, indicating no problems with multicollinearity.

Deterioration in parent‒child relationships when schools were closed was positively correlated with parents’ mental health concerns (β = 0.252, *p* = 0.005) and children’s problematic behaviors (β = 0.284, *p* = 0.002). Although deterioration in parent‒child relationships after schools reopened was positively correlated with difficulty washing hands (β = 0.253, *p* = 0.007) and problematic behaviors (β = 0.351, *p* < 0.001), it was negatively correlated with parents’ mental health concerns (β = − 0.214, *p* = 0.021).

No relevant factors were observed regarding deterioration in relationships with friends when schools were closed, but a positive correlation was observed with difficulty maintaining social distancing (β = 0.299, *p* = 0.002), difficulty washing hands (β = 0.205, *p* = 0.029), and problematic behaviors after schools reopened (β = 0.282, *p* = 0.003); a negative correlation was observed with “Voluntarily complying with stay-at-home requests” (β= − 0.211, *p* = 0.021).

**Table 5:** Multiple regression analysis results regarding the deterioration of relationships with friends

Explanatory variable	When schools were closed					After schools reopened					
	Crude B	95% CI^a^	β	95% CI	*p*-value	Crude B	95% CI	β	95% CI	*p*-value	
Social distancing	0.247	0.070–0.381	0.163	–0.012–0.311	0.069	–0.014	–0.164 − 0.139	0.018	–0.140–0.171	0.846	
Washing hands	0.032	–0.143–0.206	–0.104	–0.283 − 0.076	0.255	0.26	0.085–0.403	0.253	0.065–0.410	0.007	
Wearing a mask	0.124	–0.051–0.305	0.047	–0.131–0.227	0.596	–0.087	–0.253 − 0.084	–0.133	–0.301–0.045	0.145	
Health check	0.021	–0.167–0.207	0.038	–0.146–0.227	0.667	0.165	–0.008–0.336	–0.031	–0.214–0.152	0.737	
Airing out	0.019	–0.184–0.228	–0.025	–0.223–0.165	0.767	0.084	–0.100–0.288	0.098	–0.081–0.297	0.259	
Voluntarily complying with stay-at-home requests	0.162	–0.012–0.353	0.025	–0.156–0.209	0.774	–0.027	–0.207 − 0.147	–0.095	–0.270 − 0.081	0.288	
Parents’ mental health concerns	0.35	0.174–0.481	0.252	0.074–0.398	0.005	–0.059	–0.206 − 0.103	–0.214	–0.348–0.029	0.021	
Problematic behaviors when schools were closed	0.371	0.188–0.484	0.284	0.100–0.413	0.002	-	-	-	-	-	
Problematic behaviors after schools reopened	-	-	-	-	-	0.303	0.118–0.410	0.351	0.144–0.467	< 0.001	

*Adjusted according to age and sex

**Problematic behaviors observed when schools were closed were adopted as explanatory variables in the “when schools were closed” model, and problematic behaviors after schools had reopened were adopted as explanatory variables in the “after schools reopened” model

^a^Confidence Interval

**Table 6:** The above associations were similar when excluding children who did not attend school before the spread of infection

Explanatory variable	When schools were closed					After schools reopened				
	Crude B	95% CI^a^	β	95% CI	*p*-value	Crude B	95% CI	β	95% CI	*p*-value
Social Distancing	0.151	–0.019–0.25	0.074	–0.090–0.204	0.444	0.243	–0.062–0.365	0.299	0.102–0.424	0.002
Washing hands	–0.097	–0.225 − 0.065	–0.165	–0.295 − 0.023	0.094	0.182	0.008–0.336	0.205	0.020–0.367	0.029
Wearing a mask	0.113	–0.054–0.249	0.039	–0.129–0.196	0.682	0.001	–0.174–0.175	–0.097	–0.273 − 0.082	0.29
Health check	–0.075	–0.224 − 0.091	0.001	–0.170–0.172	0.989	–0.027	–0.207 − 0.153	0.172	–0.363 − 0.013	0.068
Airing out	0.161	–0.335 − 0.014	–0.164	–0.341 − 0.013	0.07	0.105	–0.082–0.321	0.109	–0.070–0.320	0.206
Voluntarily complying with stay-at-home requests	0.207	0.030–0.335	0.183	–0.005–0.326	0.057	–0.123	–0.301 − 0.053	–0.211	–0.392 − 0.033	0.021
Parents’ mental health concerns	0.154	–0.016–0.260	–0.128	–0.047–0.249	0.178	0.041	–0.123–0.197	–0.176	–0.324 − 0.005	0.057
Problematic behaviors when schools were closed	0.084	–0.071–0.198	0.033	–0.115–0.166	0.724	-	-	-	-	-
Problematic behaviors after schools reopened	-	-	-	-	-	0.203	0.026–0.334	0.282	0.086–0.414	0.003

*Adjusted according to age and sex

**Problematic behaviors observed when schools were closed were adopted as explanatory variables in the “when schools were closed” model, and problematic behaviors after schools reopened were adopted as explanatory variables in the “after schools reopened” model

^a^Confidence Interval

## Discussion

This study examined children with neurodevelopmental disorders and investigated (a) the association between their relationships with parents and peers and difficulty implementing infection control measures and (b) the factors related to this difficulty while the infection spreads. The results showed that 84.1% of children with neurodevelopmental disorders had difficulties implementing infection control measures, with social distancing being the most common. Difficulties in implementing infection control measures deteriorated parent and friend relationships after schools reopened.

Notably, in observing the relationships that children with neurodevelopmental disorders have with other people, this study found that the neurodevelopmental children’s parent–child relationships deteriorated when schools were closed, but did not change when schools reopened. In contrast, their relationship with friends deteriorated when schools were closed, but improvement was observed after schools reopened. These results align with the study by Hagihara et al. [8], which showed that the sense of closeness that elementary school pupils with neurotypical development felt toward their friends was higher after schools reopened than when schools were closed.

Moreover, 84.1% of the children showed some difficulties in implementing infection control measures. A survey conducted in Singapore during the implementation of intensive measures, such as the closure of schools and discontinuation of non-essential services, and immediately after showed that 50% of children with neurodevelopmental disorders had difficulty implementing infection control measures [20]. Our survey was performed several months after schools reopened; hence, along with the return to regular social life, difficulty implementing infection control measures may have become increasingly highlighted. Keeping social distance was the most common difficulty. Similar results were reported in a previous study conducted in elementary schools in the United Kingdom [22] and in a study of children with Down syndrome [23]. For school-aged children, balancing learning activities and infection control may be difficult.

Multiple regression analysis was conducted to investigate changes in relationships with other people. The results showed that difficulty implementing infection control measures was unrelated to parent‒child relationships when schools were closed; however, there was an association between parents’ mental health concerns and the child’s problematic behaviors. The association between “acting out” by the child and deterioration of parent‒child relationships has been previously noted [9], and similar tendencies were observed during the COVID-19 pandemic, when problematic behaviors of children with neurodevelopmental disorders increased [6]. Moreover, a study conducted in the USA during the COVID-19 pandemic reported that parent‒child relationships were affected by the caregivers’ mental health concerns [12]. In Japan, where lockdowns were not imposed and relatively loose regulations were applied, the caregivers’ mental health concerns still influenced parent‒child relationships.

In contrast, difficulty in implementing infection control measures was linked to parent‒child relationships after schools reopened. With the spread of COVID-19, infection control activities have become essential skills for daily life. Difficulty practicing daily living skills, including repeated handwashing at home, increased parents’ burdens in raising their children [24] and may have impacted parent‒child relationships.

Our study also showed that problems with caregivers’ poor mental health after school reopened had an inverse relationship with deteriorating relationships with their children. Caregivers’ mental health conditions are related to increased negative interaction with children during the COVID-19 pandemic [25]. The reduction of time spent at home by school reopening may ameliorate their deteriorating relationships.

No factors were observed to be related to the deterioration of relationships with friends when schools were closed. In contrast, of the difficulties in implementing infection control measures, those related to social distancing and washing hands were significantly related to the deterioration of relationships with friends after schools reopened. When schools reopened, interactions with friends increased. Infection control was emphasized at school, similar to at home. Partly because of changes in the environment, the child’s difficulties in implementing infection control measures may also be related to deterioration in their involvement with friends. In contrast, difficulty in voluntarily complying with stay-at-home requests was linked to improved relationships with friends. The more difficult a child found complying with staying at home, the greater the opportunities may have been to go out after schools reopened, increasing their interactions with friends.

An increase in problematic behaviors was also related to the deterioration of children’s interaction with friends after schools reopened. This result was similar to the result of a previous study that observed an association between children’s relationships with friends and their problematic behaviors [14].

This study has some limitations. The questionnaire used was developed for this study and has not been examined for reliability. Retrospectively conducting a questionnaire survey with caregivers may have affected their assessment of their child’s relationship with other people. Specifically, a child’s relationship with friends is an area that may not be wholly known to caregivers. In future research, by combining the evaluation methods of friendships by a child and his or her friends, it will be possible to grasp the situation of more realistic friendships. Moreover, although this study examined changes in a child’s relationship with their caregiver and friends, it did not consider the presence of siblings, who are “important others” to the child. Therefore, it is necessary to also investigate changes in sibling relations. Finally, the literature and the current study do not provide evidence regarding when and how support for infection control measures is useful for children with neurodevelopmental disorders. Duncan [26] states that strategies such as visual support, task analysis, technology (e.g., smartphone apps), modeling, and explicit and constructive feedback are useful in programs for acquiring skills in daily life, including hand washing for adolescents with autism spectrum disorder. A future, objective is hoped to develop methods for the acquisition of infection control skills incorporating these strategies that can be used at home and in schools in case the event of similar pandemics.

## Conclusion

In this study, the relationships between children with neurodevelopmental disorders and the difficulties in infection control during the pandemic were examined. Difficulties in infection control as a factor related to the deterioration of relationships with others was newly considered. The difficulty experienced by children with neurodevelopmental disorders in implementing infection control measures affected their relationships with parents and peers after schools reopened and children returned to social life. Infection prevention measures remain ongoing. Therefore, changes in the relationships of children with neurodevelopmental disorders with their parents and friends must be closely monitored as they continue to live in group settings.

## Electronic supplementary material

Below is the link to the electronic supplementary material.

Supplementary Material 1

## Data Availability

The datasets used and/or analyzed during the current study are available from the corresponding author on reasonable request.
