# Supplementary material for: Relationship between children with neurodevelopmental disorders and their caregivers and friends during early phase of COVID-19 school closure in Japan: Association with difficulty in implementing infection prevention measures
Source: Child Adolesc Psychiatry Ment Health. 2022 Oct 7;16:78. doi: 10.1186/s13034-022-00513-7 (PMC9542459; doi:10.1186/s13034-022-00513-7)
Supplement: Supplementary file 1 — Supplementary Material 1 [file 13034_2022_513_MOESM1_ESM.pdf]

# Questionnaire for the survey on the situation during the spread of COVID-19 infection

## 1. Demographic Data

① Prefecture/district that you live in ________________

② How old is your child? ________________Years ____________ months

③ Gender male/ female

④ Which diagnosis does your child have?

☐Autism Spectrum Disorders

☐ADHD

☐Learning Disability

☐Intellectual disability

☐others（ ）

⑤ Family members living with your child

☐Father

☐mother

☐grandfather

☐grandmother

☐others

☐Siblings : 1- _____(age) _______________

2- _____(age)________________

3- _____(age)________________

4- ____ (age)_________________

Any of the siblings has medical problems or developmental disorders? If Yes;

①_________years ②___________years

③ _________years ④___________years

⑥ Do your child go to center of intervention? Yes / No

⑦ What kind of school does your child go to?

☐Public elementary primary school

☐Private elementary primary school

☐Public junior high school

☐Private junior high school

☐Special school

☐Others（ ）

⑧ Did your child have online classes during declaration of a state of emergency

Yes/ No

⑨ Please fill in the bar about your child’s activities during declaration of a state of emergency.

E.g. Bedtime, wake up time, meal time, bathing time.

| 0 | 1 | 2 | 3 | 4 | 5 | 6 | 7 | 8 | 9 | 10 | 11 | 12 | 13 | 14 | 15 | 16 | 17 | 18 | 19 | 20 | 21 | 22 | 23 | 24 | o’clock |
|---|---|---|---|---|---|---|---|---|---|---|---|---|---|---|---|---|---|---|---|---|---|---|---|---|---|
| | | | | | | | | | | | | | | | | | | | | | | | | | |

⑩ What were the restrictions or the voluntary ban during declaration of a state of emergency.

☐ My child has been unable to go to school

☐ My child has been unable to go to the center of intervention.

☐ My child has been unable to play or exercise outside

☐ My child has been unable to go to shopping

☐ No outdoor activities were permitted, he/she was confined to the home

☐ Restrictions affecting access to non-urgent medical care

☐ Restrictions affecting access to urgent/emergency medical care

☐ Break from normal routine eg: online based therapy

☐ others（ ）

⑪ Where is your child in the daytime during declaration of a state of emergency?

☐Home

☐Afterschool child care

☐Afterschool daycare

☐School

☐House of relatives

☐Other（　　　　　　　　）

⑫ About the father of your child

- The father is not in contact with the family
- Employment status:

Employed/ unemployed

→Which working pattern during declaration of a state of emergency if a father was in employment?

☐Working at the office

☐Telework/Work from home

☐A temporary layoff

☐Paid holiday

☐Other

⑬ About mother of your child

- The mother is not in contact with the family
- Employment status:

Employed / Unemployed

→Which working pattern during declaration of a state of emergency if a mother was in employment?

☐Working at the office

☐Telework/Work from home

☐A temporary layoff

☐Paid holiday

☐Other

⑭ Did anyone screen positive for COVID-19 (your child, close family members)?

Yes / No

→If Yes, Who was / were screened positive for COVID-19?

☐Your child

☐Families living together

☐Families living apart

☐Your child’s friend

☐Acquaintance

⑮ What type of house do you live in?

house ・ collective housing ・ others（ ）

⑯ Do you have a yard or balcony?

Yes/ No

2. About your child's relationship with friends during declaration of a state of emergency

1. Did your child play or contact a schoolmate in any way? yes/no

(If yes) How did your child get involved with his friends? How many times did he/she do this?

☐ Playing directly (about __time(s))

☐ Talking on the phone (about __time(s))

☐ Sending messages with a messaging app (ex:LINE) (about __time(s))

☐ Playing online games together (about __time(s))

☐ other

(When your child was socializing with friends over the phone, Line, or online games) Which of the following were problems during the declaration of a state of emergency?

☐ Communication through the phone didn't go well

☐ Your child made long calls

☐ One-sided talk on the phone

☐ Communication over LINE didn't go well

☐ It was difficult to follow the rules, such as continuing the communication on LINE until late.

☐ He/she made an inappropriate statement on Group LINE.

☐ Communication through internet games didn't go well

☐ It was difficult to follow the rules, such as limiting his/her internet games time.

(If No) How was your child's interest in his/her friends during declaration of a state of emergency?

☐ He/she had no interest.

☐ He/she was comfortable that there was no contact with friends.

☐ He/she seemed to want to communicate with his/her friends, but didn't try to do anything to interact.

☐ He/she tried to communicate, but it didn't work.

☐ He/she communicated with a friend not from his/her school.

☐ He/she was communicated with in someone/several people who were not real friends, through online games

## 3. About your child's behavior and relationship

（1）Has your child had any problems during the period prior to the COVID-19 pandemic, during declaration of a state of emergency or after school restarted. Check the box if any.

| | The existing problems before pandemic | During declaration of a state of emergency | | After school restarted | |
|---|---|---|---|---|---|
| | | Worsening of exiting problems | New problems | Worsening of exiting problems | New problems |
| Sleep issues | | | | | |
| Food refusal | | | | | |
| Hyperactivity | | | | | |
| Inattention | | | | | |
| Oppositional behavior | | | | | |
| Irritability | | | | | |
| Withdrawing from other people | | | | | |
| Temper tantrums | | | | | |
| Aggression | | | | | |
| Self-injurious behavior | | | | | |
| Humming, any other unusual noises(Repetitive noise) | | | | | |
| Unusual repetitive movements (i.e.: jumping on the spot, flapping of hands, etc.) | | | | | |
| Obsessions | | | | | |
| School refusal | | | | | |
| Other( ) | | | | | |

② How were your child’s relationships with friends?

a) Prior to the pandemic

good / fairly good / neither poor nor good / fairly poor / poor

b) During declaration of a state of emergency

good / fairly good / neither poor nor good / fairly poor / poor

c) After school restarted

good / fairly good / neither poor nor good / fairly poor / poor

③ How is your child’s relationship with parents?

a) Prior to pandemic

good / fairly good / neither poor nor good / fairly poor / poor

b) During declaration of a state of emergency

good / fairly good / neither poor nor good / fairly poor / poor

c) After school restart

good / fairly good / neither poor nor good / fairly poor / poor

④ How is your child’s relationship with other siblings?

a) Prior to pandemic

good / fairly good / neither poor nor good / fairly poor / poor

b) During declaration of a state of emergency

good / fairly good / neither poor nor good / fairly poor / poor

c) After school restart

good / fairly good / neither poor nor good / fairly poor / poor

## 4. About your child's infection control measures

1.Which infection control measures were difficult to do well for your child?

☐ Refraining from going out

☐ Keeping a distance of 2 meters from people

☐ Wearing a mask when you out/indoors or having a conversation

☐ Making sure your child washes his/her hands carefully, when arriving home

☐ Taking your child's temperature and checking your child's health in the morning

☐ other

2.Why was it difficult for your child?

☐ It was difficult for my child to understand the necessity of it

☐ It was difficult because of hypersensitivity

☐ It was difficult for my child to understand the abstract concept (e.g. Physical condition, social distance)

☐ My child refused the infection control measures for unknown reasons

☐ My child forgot to do these, though he understood the need to do so.

☐ It was difficult because of obsessive behaviour

☐ My child did practiced these measures too much

☐ other

## 5. About family members during the declaration of a state of emergency

① Did any of your family members who stayed together face psychological problems during the declaration of a state of emergency?

Yes/ No

(If Yes)

Who? Father/ mother/ siblings/ other family members

What were the psychological issues?

☐ Emotional stress

☐ Anxiety

☐ Depression

☐ Others: ________________________________________________________________

② What bothered you about your child during the declaration of a state of emergency?

☐ Getting him/her to study (e.g. school homework)

☐ Getting him/her to do what he/she needed to do

☐ Getting him/her to utilise free time

☐ Limiting his/her time watching TV, using a smartphone or playing a game with a digital device.

☐ Bad relationship between him/her and his/her sibling(s) (e.g. a fraternal squabble)

☐ Clinging to me

☐ Dealing with the complaint of anxiety

☐ Dealing with the his/her irritability

☐ Dealing with the his/her restricted, repetitive behavior, interest or activity

☐ Being forced into his/her restricted, repetitive interest or activity

☐ Getting him/her to wait while I did what I needed to do (e.g. telework or housekeeping)

☐ For no special reason, but I felt it was hard.

③ Did you have any support (e.g. Consultation, rehabilitation) during declaration of a state of emergency?

Yes / No

④ What kind of support did you receive during declaration of a state of emergency?

☐ Consultation on child development and worried behavior

>How? Online / face-to-face / phone

>By whom? Your family / Your friend / School teacher / School psychologist / Doctor / Psychologist in hospital / therapist (ST, OT, other therapist) / Others

☐ Counseling about your psychological problems

>How? Online / face-to-face / phone

>By whom? Your family / Your friend / School teacher / School psychologist / Doctor / Psychologist in hospital / therapist (ST, OT, other therapist) / Others

☐ Counseling or psychological therapy for child directly

>How? Online / face-to-face / phone

＞By whom？ Child’s family / Child’s friend / School teacher / School psychologist / Doctor / Psychologist in hospital / therapist (ST, OT, other therapist) / Others

☐ Rehabilitation for child directly

>How? Online / face-to-face / phone

☐ Consultation on medication for problem behavior of your child

>How? Online / face-to-face / phone

☐ Support from school

>How? Online / face-to-face / phone

☐ Daytime childcare

>Where? afterschool childcare / afterschool daycare / respite care / House of family who lives apart (e.g. grandparents' homes) / other( )

☐ Someone who helps with child-raising at home

>Who? Family / Family living apart / Babysitter / Public family support system /other( )

☐ Someone who helps with housework

>Who? Family / Family living apart / Homemaking service / other( )

⑤ What kind of support did you want?

☐ Consultation on child development and worried behavior

>How? Online / face-to-face / phone

>By whom? Your family / Your friend / School teacher / School psychologist / Doctor / Psychologist in hospital / therapist (ST, OT, other therapist) / Others

☐ Counseling about your psychological problems

>How? Online / face-to-face / phone

>By whom? Your family / Your friend / School teacher / School psychologist / Doctor / Psychologist in hospital / therapist (ST, OT, other therapist) / Others

☐ Counseling or psychological therapy for child directly

>How? Online / face-to-face / phone

＞By whom？ Child’s family / Child’s friend / School teacher / School psychologist / Doctor / Psychologist in hospital / therapist (ST, OT, other therapist) / Others

☐ Rehabilitation for child directly

>How? Online / face-to-face / phone

☐ Consultation on medication for problem behavior of child

>How? Online / face-to-face / phone

☐ Support from school

>How? Online / face-to-face / phone

☐ Daytime childcare

>Where? afterschool childcare / afterschool daycare / respite care / House of family who lives apart (e.g. grandparents' homes) / other( )

☐ Someone who helps with child-raising at home

>Who? Family / Family living apart / Babysitter / Public family support system /other( )

☐ Someone who helps with housework

>Who? Family / Family living apart / Homemaking service / other( )

⑥ Why did you select the above choices?

- ☐ Expertise in developmental disorders
- ☐ Low risk of infection
- ☐ Familiar with parents or child
- ☐ Low financial burden
- ☐ others

Thank you for your cooperation.
